# Interaction of letrozole and its degradation products with aromatase: chemometric assessment of kinetics and structure-based binding validation

**DOI:** 10.1080/14756366.2022.2081845

**Published:** 2022-05-29

**Authors:** Michele De Luca, Maria Antonietta Occhiuzzi, Bruno Rizzuti, Giuseppina Ioele, Gaetano Ragno, Antonio Garofalo, Fedora Grande

**Affiliations:** a Department of Pharmacy, Health and Nutritional Sciences, University of Calabria, Rende, Italy; b CNR-NANOTEC, SS Rende (CS), Department of Physics, University of Calabria, Rende, Italy; c Institute for Biocomputation and Physics of Complex Systems (BIFI), Joint Unit GBsC-CSIC-BIFI, University of Zaragoza, Zaragoza, Spain

**Keywords:** Breast cancer, drug stability, multivariate curve resolution, spectrophotometry, molecular docking, enzyme inhibition

## Abstract

Letrozole is one of the most prescribed drugs for the treatment of breast cancer in post-menopausal women, and it is endowed with selective peripheral aromatase inhibitory activity. The efficacy of this drug is also a consequence of its long-lasting activity, likely due to its metabolic stability. The reactivity of cyano groups in the letrozole structure could, however, lead to chemical derivatives still endowed with residual biological activity. Herein, the chemical degradation process of the drug was studied by coupling multivariate curve resolution and spectrophotometric methodologies in order to assess a detailed kinetic profile. Three main derivatives were identified after drug exposure to different degradation conditions, consisting of acid-base and oxidative environments and stressing light. Molecular docking confirmed the capability of these compounds to accommodate into the active site of the enzyme, suggesting that the sustained inhibitory activity of letrozole may be at least in part attributed to the degradation compounds.

## Graphical Abstract

**Figure:**
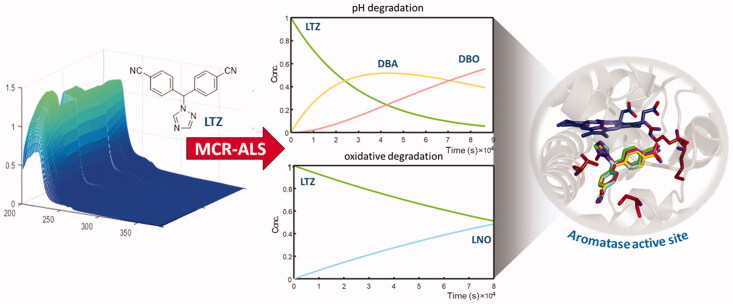

## 1. Introduction

The emergence and progression of hormone-responsive breast cancer largely depend on the endocrine oestrogenic activity. The treatment of this form of cancer is currently pursued following two main approaches. The first one, more effective during pre-menopausal events when the gonads are the primary producers of oestrogen, is based on the use of antagonists targeting oestrogen receptors. In this case, tamoxifen is the most prescribed drug^1^. A second approach is preferred in post-menopause, when the production of oestrogen is limited to extra-glandular tissues and consists in the administration of aromatase inhibitors. This enzyme, belonging to the CYP450 superfamily, is expressed in several tissues including gonads, placenta, brain, bone, and adipose tissue. Aromatase acts as a monooxygenase, catalysing the conversion of androgen to oestrogen; thus, its inhibition results in a near complete oestrogen deprivation. Several compounds have been proposed as aromatase inhibitors, leading to a first, second, and third generation of specific agents, the latter being the most prescribed nowadays. Fadrozole and vorozole, which show a non-steroidal structure, were once commonly prescribed but are no longer utilised today, whereas the use of the irreversible steroid inhibitor exemestane is limited to patients pre-treated with tamoxifen in adjuvant therapy^2^^,^^3^.

**Figure 1.**
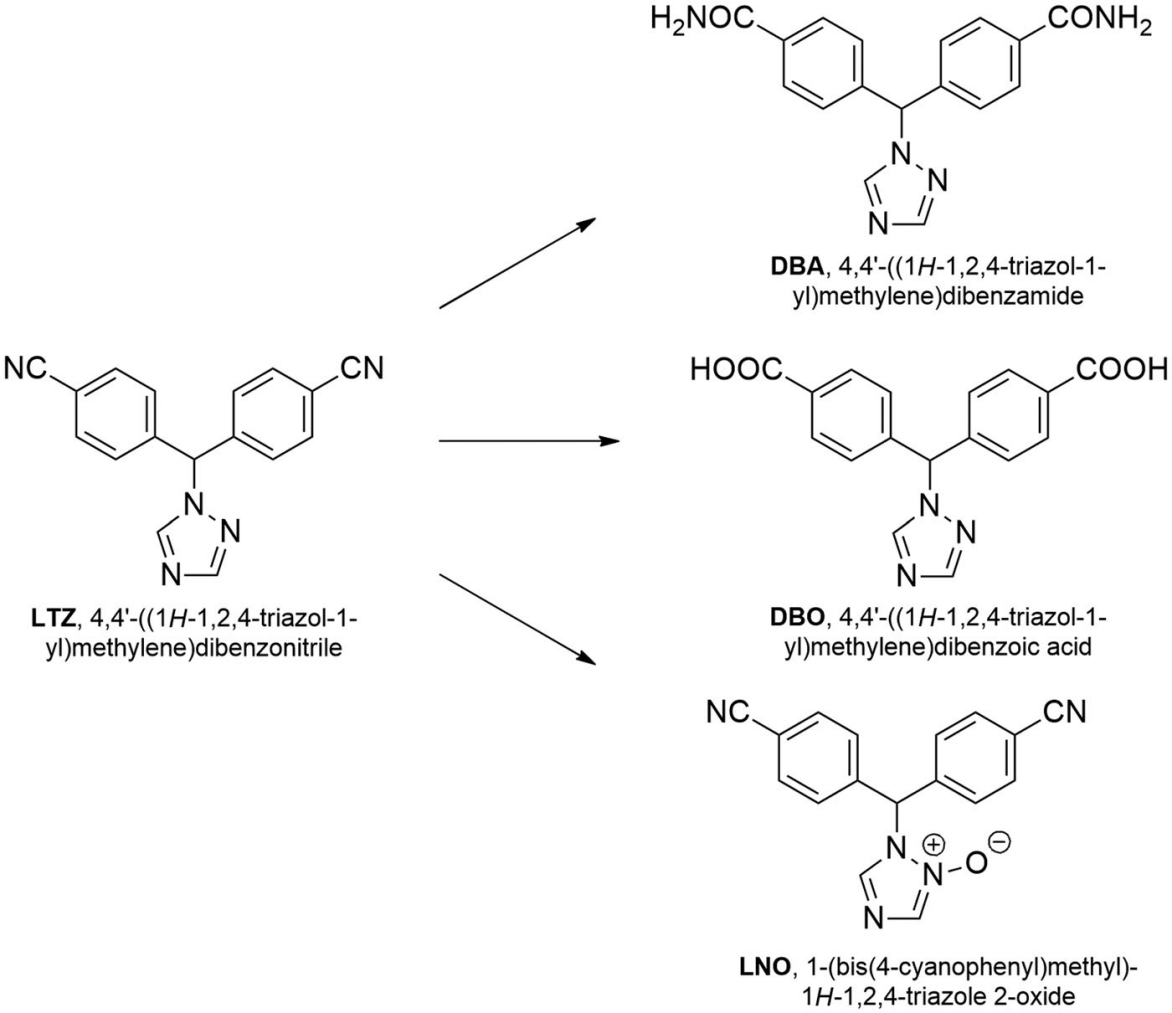
Chemical structure of LTZ and its degradation products.

Letrozole (LTZ) and anastrazole, two compounds showing structural similarities, are today the most used reversible non-steroidal inhibitors^4^. The scaffold of both LTZ and anastrazole is based on a 1,2,4-triazole ring substituted in position 1 with different benzylic residues bearing two cyano groups, whose presence seems necessary for the anti-enzymatic activity. These peculiar features suggest a considerable chemical reactivity, which has been studied under different conditions^5^^,^^6^. In particular, LTZ is slightly more potent and longer-acting than anastrazole, and has been shown to easily undergo hydrolysis of its cyano groups in both acidic and alkaline medium, resulting in the formation of the corresponding dicarboxylic acid or dicarboxyamide, respectively (Figure 1). On the other hand, under oxidising condition, the N-oxide in position 2 of the triazole was the main product generated. These three main degradation products have not yet been fully studied with regard to their intrinsic inhibitory activity, which could lead to a prolongation of the drug action *in vivo*.

Previous studies have shown that LTZ is highly sensitive to extreme pH values and oxidative conditions, whereas it is stable under light irradiation^5–7^. Many experimental studies have been focussed on LTZ and its metabolites or degradation products, which have been investigated by various analytical techniques. The chromatographic approach was successfully applied using different detector systems such as UV/Vis, fluorescence, and mass spectroscopy^5^^,^^8–10^. Spectroscopic methodologies were also used for assaying the drug in pharmaceutical formulations^11–13^.

In this work, we describe in detail the degradation profile of LTZ under different experimental conditions. It is well known that nitrile catabolism can follow two distinct pathways: (1) conversion to carboxylic acid catalysed by nitrilase, and (2) amide formation mediated by nitrile hydratase/amidase. These reactions can be easily reproduced using different hydrolytic chemical conditions^14^. A further factor leading to LTZ degradation is represented by non-hydrolytic oxidative conditions, when an easy formation of the corresponding 1-(bis(4-cyanophenyl)methyl)-1*H*-1,2,4-triazole-2-oxide (LNO) is observed. The formation of other minor degradation products has been reported^6^, but their presence has not been considered here. This study aimed to define the kinetics of the degradation products of LTZ, monitoring them by UV/Vis spectrophotometry and processing the spectral data by Multivariate Curve Resolution-Alternating Least Squares (MCR-ALS) methodology. This chemometric procedure was used because it is particularly effective in following the chemical transformation processes, allowing to resolve the spectra and concentration profiles of the components involved. An independent HPLC-DAD method was defined to validate the results obtained by the multivariate resolution of the UV kinetic studies. The UV spectra from the HPLC-DAD detector were compared with the spectra predicted by the multivariate procedure, demonstrating a significant overlap of the spectral curves related to the degradation products. Furthermore, docking experiments were performed to verify whether the degradation compounds were able to accommodate in the aromatase active site with binding mode conformation and affinity comparable to the parent drug. To this aim, LTZ and its three main degradation compounds were docked in the crystal structure of aromatase and the results were compared with those obtained for androstenedione (ASD), the endogenous ligand of the enzyme.

## 2. Materials and methods

### 2.1. Chemicals

All samples were prepared using distilled water and analytical grade reagents. LTZ standard was purchased from Merck (Milan, Italy), with certified purity >98%, and was used as received. Hydrochloric acid, sodium hydroxide, hydrogen peroxide, HPLC grade water and acetonitrile were also supplied by Merck (Milan, Italy).

### 2.2. Instruments

A Crison pH-meter GPL 22 (Barcelona, Spain) was used to measure the pH values of the samples during degradation. Photodegradation experiments were carried out in a chamber Suntest CPS+ (Atlas, Milan, Italy), equipped with a xenon lamp, using light irradiation according to the ID65 standard of the ICH rules (International Council for Harmonisation of Technical Requirements for Pharmaceuticals for Human Use)^15^. Spectrophotometric measurements were recorded using an Agilent 8453 Diode Array spectrophotometer (Agilent Technologies, CA, USA). HPLC analysis was performed by using an Agilent 1100 series chromatograph (Agilent Technologies, CA, USA), equipped with a binary pump delivery system and a diode array UV–Vis detector.

Spectrophotometer ChemStation software (Agilent Technologies, CA, USA) was used for acquiring experiment data and converting raw UV spectra files (.sd) to human-readable files (.csv), suitable to be imported directly into MATLAB® (The MathWorks, Inc., MA, USA). All chemometric analyses were performed under MATLAB^®^ computing environment, where the MCR-ALS procedure (GUI version 2.0) has been implemented^16^.

### 2.3. Experimental procedures

A standard stock solution of LTZ (1 mg/mL) in ethanol was properly diluted to obtain seven samples (10 µg/mL) at pH values close to 1, 3, 5, 7, 9, 10, 12 by addition of HCl or NaOH. The pH adjustment was verified not to cause any significant dilution. These samples, transferred in perfectly stoppered quartz cells, were monitored by UV/Vis spectrophotometric analysis in the wavelength range 220–400 nm, starting just after sample preparation (t = 0 min) and every 10 min, up to 24 h.

A second sample set was prepared by combining different pH values and oxidative conditions. Appropriate aliquots of 30% (v/v) H_2_O_2_, HCl and NaOH were added to the LTZ stock solution to obtain three LTZ samples with concentration 10 µg/mL containing 0.5% (v/v) of H_2_O_2_ at pH values close to 2, 7 and 12. These samples were monitored by UV/Vis analysis (200–400 nm) every 10 min, up to 24 h.

Photolability of LTZ was tested by exposing the LTZ sample (10 µg/mL) to a xenon lamp under the following conditions: light radiation between 300 and 800 nm, power 550 W/m^2^ (33 kJ/(min x m^2^), constant temperature of 25 °C^15^. LTZ behaviour to light exposure was monitored by recording UV spectra at time t = 0 min and every 10 min along the photodegradation experiments, up to 12 h.

HPLC analysis was performed on a reverse phase Gemini LC column (250 × 4.60 mm, 5 μm C18, Phenomenex, Torrance, CA) using phosphate buffer (pH 5.8) and acetonitrile in the ratio 80:20 (v/v) as mobile phase, pre-filtered through a 0.45 µm filter. The injection volume was 20 µL and the flow rate of the mobile phase was 1.0 ml/min at room temperature of 20 °C. HPLC was performed on LTZ ethanol solutions (1 mg/mL) exposed to hydrolytic degradation with HCl and NaOH and oxidation with H_2_O_2_ immediately after preparation (t = 0 min) and after 24 h.

### 2.4. Multivariate curve resolution – alternating least squares (MCR-ALS)

Multivariate Curve Resolution (MCR) defines a family of methodologies aimed at resolving the chemical contributions to the outcome of an experiment, described through a data matrix. They have been applied to study different types of multivariate or multicomponent chemical systems^17^. MCR method decomposes the experimental data matrix (**D**) into a reduced set of contributions of chemical species (in our study, LTZ and its degradation products), using a bilinear model obtained rewriting the Lambert-Beer’s law in multiway mode^17^^,^^18^:

$$D=CS^{T}+E$$

where **D** (n,m) is the experimental data matrix, **C** (n,k) is the matrix that includes the concentration evolution of the *k* components, **S**^T^ (k,m) represents the pure spectra of *k* species, and **E** (n,m) is the unexplained variance in the model.

The number of components involved in the matrix D (chemical rank) can be estimated by Principal Component Analysis (PCA) or Singular Value Decomposition (SVD) algorithms^19^. The chemical rank assumes that the species contributing to the measured spectra give singular values larger than the other signal contributions, such as experimental or instrumental noise. A rank deficiency may occur in some cases, leading PCA to identify a chemical rank lower than the actual value, but multi-experiments and the subsequent matrix augmentation obtained by processing all experiments can be used to avoid this drawback^16^. MCR allows simultaneous processing of data from different origins, via row-wise, column-wise, or both row- and column-wise data matrix augmentation.

Once the number of components is known, the ALS iterative algorithm uses a preliminary estimation of **S^T^** or **C** matrices, and a series of constraints to optimise the MCR model. The application of constraints such as non-negativity, unimodality, and concentration closure allows one to optimise the results according to a chemical meaning.

The quality and reliability of the multivariate resolution can be assessed using the explained variance (%R^2^) and the lack of fit (%)^20^. These figures of merit allow to evaluate the dissimilarity between the experimental matrix **D** and the data modelled by MCR-ALS elaboration. The equations are listed below:

$$\text{Lak}\;\text{of}\;\text{fit}\;(\%)=100\sqrt{\frac{\sum_{i,j}e_{ij}^{2}}{\sum_{i,j}d_{ij}^{2}}}$$

$$R^{2}=100\sqrt{\frac{\sum_{i,j}d_{ij}^{2}-\sum_{i,j}e_{ij}^{2}}{\sum_{i,j}d_{ij}^{2}}}$$

where *d_ij_* is an element of the matrix **D** and *e_ij_* is the related residual value obtained from the difference between the matrix **D** and the calculated **CS^T^** matrix.

In the present work, two different MCR-ALS techniques were applied to the datasets. The standard soft-MCR-ALS (S-MCR-ALS) was applied for the preliminary analysis of all data matrices and the hard and soft MCR-ALS (HS-MCR-ALS) was used to analyse in depth the degradation kinetics.

### 2.5. Molecular docking

Molecular docking was performed starting from a crystallographic structure of aromatase. The Protein Data Bank (PDB) contains 38 structures of aromatase and, among them, 27 belong to *Homo sapiens*. The entry selected was the one recently deposited with PDB ID: 5JKV^21^, and it was chosen over the others because it has a more complete amino acid sequence and contains the endogenous ligand ASD in the binding site. It also contains in a secondary allosteric binding sites a molecule of pentaethylene glycol (PEG), which acts as a weak inhibitor of aromatase. Both ASD and PEG were removed from the protein, whereas the haem cofactor was left in its position. The molecular structures of the ligands (LTZ, DBA, DBO, LNO; see again Figure 1) were built by using the modelling software Avogadro^22^.

Docking calculations were performed by using AutoDock Vina 1.1.2^23^. Preliminary conversion of the structures from the PDB format was carried out by using the graphical interface AutoDock Tools 1.5.6^24^. During the conversion, polar hydrogens were added to the crystallographic enzyme structures, whereas apolar hydrogens of the ligands were merged to the carbon atom they are attached to. Full flexibility was guaranteed for the ligands, resulting in 3, 5, 7 and 3 rotatable dihedral angles for LTZ, DBA, DBO and LNO, respectively. A single simulation run was carried out in each case at very high exhaustiveness, 16 times larger than the default value^25^^,^^26^. The binding modes of the ligands were analysed through visual inspection, and intermolecular interactions were evaluated by using the automated protein-ligand interaction profiler PLIP^27^.

## 3. Results and discussion

### 3.1. Kinetic analysis of LTZ in degradation experiments

Spectral data recorded during the degradation experiments were arranged in eleven data subsets, seven matrices **D_pHi_** of size 144 × 181 (i = 1, 3, 5, 7, 9, 10, 12) from acid-base degradation experiments, three matrices **D_oxj_** (144 × 181; j = 2, 7, 12) from oxidative degradation, and the matrix **D_λ_** (72 × 181) from the photodegradation experiment.

Initially, S-MCR-ALS analysis was performed for each experiment by defining two augmented matrices from **D_pHi_** and **D_oxj_** subsets and the single matrix **D_λ_**.

**Figure 2.**
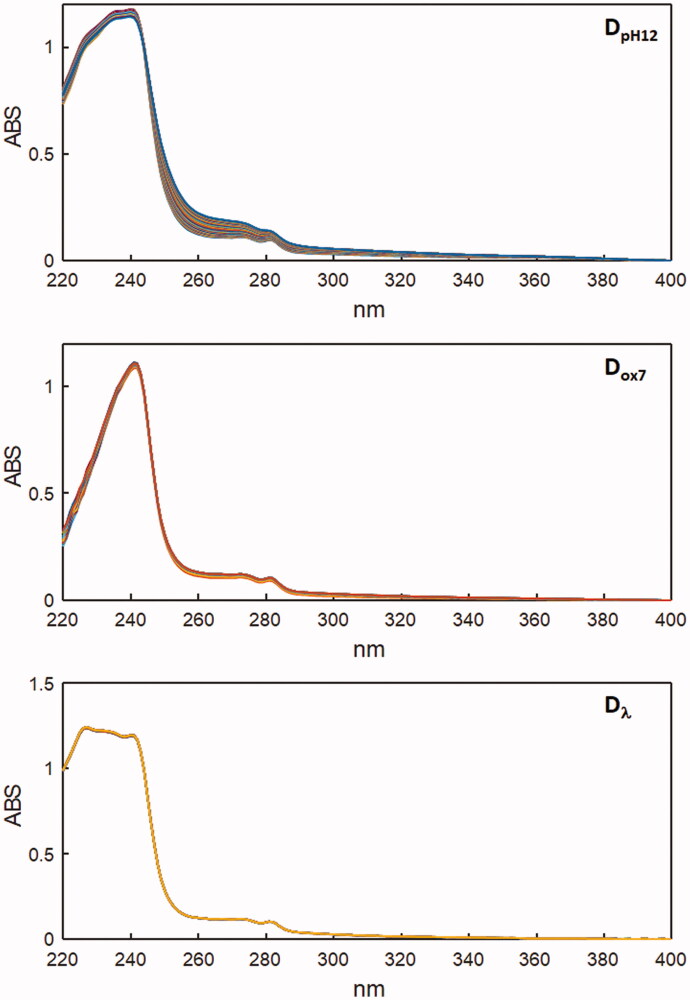
Degradation spectral data recorded in **D_pH12_**, **D_ox7_** and **D_λ_** matrices.

Augmented matrices **D_pHaug_**= [**D_pH1_**;**D_pH3_**;**D_pH5_**;**D_pH7_**;**D_pH9_**;**D_pH10_**;**D_pH12_**] = [**C_pH1_**;**C_pH3_**;**C_pH5_**;**C_pH7_**;**C_pH9_**;**C_pH10_**;**C_pH12_**] [**S_pH_^T^**] and **D_oxaug_**= [**D_ox1_**; **D_ox7_**; **D_ox12_**] = [**C_ox1_**;**C_ox7_**;**C_ox12_**] [**S_ox_^T^**] were built by merging data subsets in column-wise mode, considering the data obtained in different experiments monitored by the same analytical procedure. The rank analysis and soft modelling of the UV data resolved four components in the acid-base degradation process, and three components in the oxidative degradation. A fair stability of the drug in solution was observed when it was exposed to light for up to 360 min, as evident in Figure 2, because the spectral signal did not undergo significant changes.

**Figure 3.**
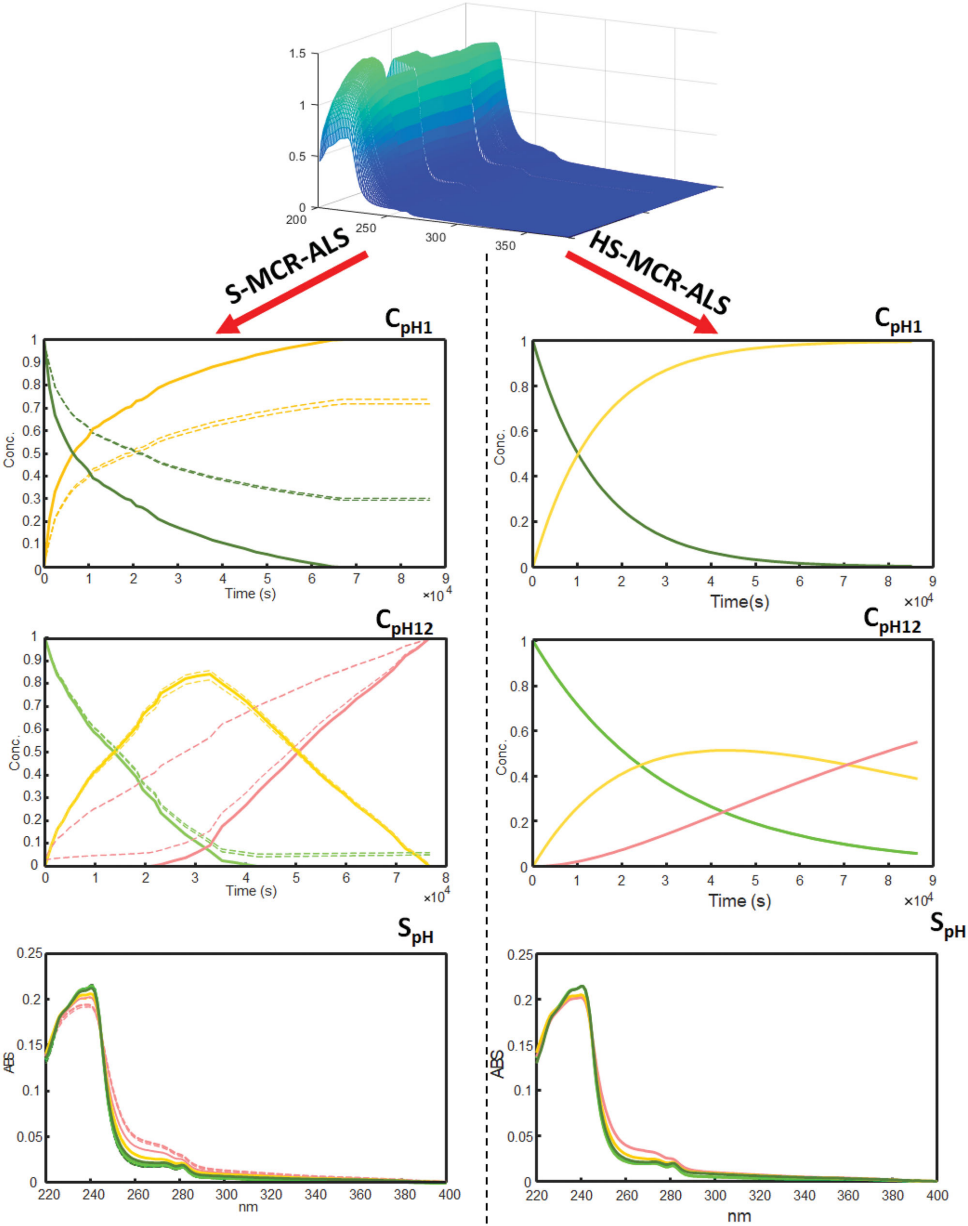
Concentration profiles and pure spectra obtained by S-MCR-ALS (- - band boundaries) and HS-MCR-ALS analysis on augmented matrix **D_pHaug_**: LTZ_a_ (dark-green), LTZ_b_ (light-green), DBA (yellow) and DBO (pink).

The rank analysis of matrix **D_pHaug_** estimated 4 species distributed within all subsets. **C_pHaug_** and **S_pHaug_^T^** were optimised by the S-MCR-ALS algorithm with satisfying data fitting in term of %LOF = 1.45% and R^2^ = 99.9%. The band boundaries of feasible S-MCR solutions were evaluated by using the MCR-BANDS routine contained in MCR-GUI^18^. The band boundaries of feasible S-MCR-ALS solutions evaluated for LTZ acid-base degradation experiment are shown in Figure 3. The rotational ambiguities were evident in matrix **C** and **S^T^** for all resolved species. The matrices **C_pHaug_** and **S_pHaug_^T^** described the concentration evolution during the degradation process (time direction) and the spectral features correlated to the species involved in the experiments arranged in **D_pHaug_**. In the acid-base degradation process, two species were resolved for LTZ at time 0, depending on the different pH. LTZ is characterised by a pK_a_ = 2.17 and two forms, the ionised LTZ_A_ and the non-ionised LTZ_B_, were detected with different spectral shape in **S_pH_^T^** (Figure 3). **C_pHaug_** resolved the component corresponding to the first degradation product at acidic pH values, which formed due to the partial hydrolysis of the cyano moiety to amide DBA (**C_pH1_**). The hydrolysis process was pH-dependent and occurred only at extreme acidity values. The drug was moderately stable close to neutral pH conditions, as no significant spectral variations were observed at pH 5, 7 and 9. In contrast, a complete hydrolysis from cyano to carboxylic groups was obtained at very high pH values. In the experiment at pH 10, only the signal corresponding to DBA was resolved, whereas at pH 12 both the amide and carboxylic hydrolysis products DBA and DBO were detected (**C_pH12_**). The acid-base degradation process of LTZ was found to follow two sequential first-order reactions at different pH values, and the proposed reaction process is LTZ > DBA > DBO (see again Figure 3).

**Figure 4.**
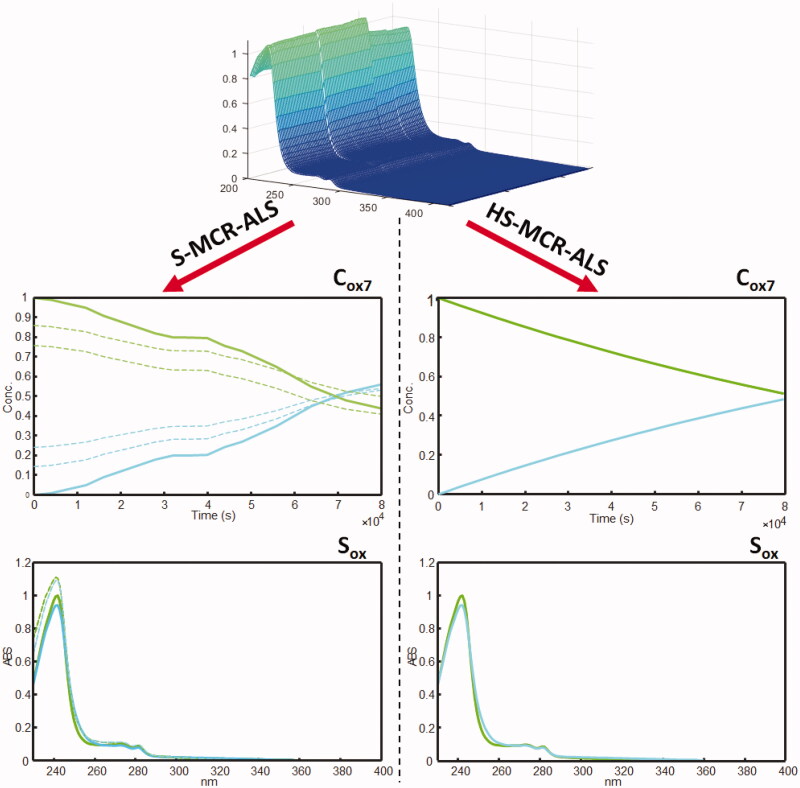
Concentration profiles and pure spectra obtained by S-MCR-ALS (- - band boundaries) and HS-MCR-ALS analysis on augmented matrix **D_oxaug_**: LTZ (green) and LNO (cyan).

The matrices **C_oxaug_** and **S_oxaug_^T^** described a different behaviour of LTZ when exposed to an oxidative environment. MCR-ALS analysis of the matrix **D_oxaug_** (% LOF = 0.87% and R^2^ = 99.9%) resolved three components, which encompassed both forms of LTZ (LTZ_A_ and LTZ_B_) and the oxidation product was characterised by the N-oxide at position 2 in the triazole of LNO, obtained from a first-order reaction LTZ > LNO (Figure 4). The different evolution of the chemical species in the various experiments highlighted the dependence of the oxidation process on the pH.

In some cases, MCR processing can be limited by the failure to guarantee a unique result, as the technique suffers from some intensity and/or rotational ambiguity (Figures 3 and 4). In the case of kinetic studies, the scale of **C** or **S^T^** is known, and the total mass balance within the system is constant. In a closed reaction kinetic system, intensity ambiguity can be solved by a normalisation procedure or applying the closure constraint. In case of rotation ambiguities, the adoption of more constraints to MCR modelling can be useful in reducing them. Aside from the natural constraints, such as no-negative, unimodality and closure constraints, the most effective tools for reducing rotation ambiguities are the use of local rank and/or selective constraints, the simultaneous analysis of multi-datasets and the use of hard modelling routines^18^.

In a subsequent step, matrices **D_pHaug_** and **D_oxaug_** were processed by HS-MCR-ALS, which was used to analyse in depth the kinetic degradations. HS-MCR-ALS uses a non-linear curve fitting routines to iteratively fit the concentration profiles resolved by MCR-ALS with the optimal kinetic rate, leading to an improved resolution of the concentration profiles of the species formed during the acid-base and oxidation experiments. It also reduces the rotational ambiguities associated with the S-MCR-ALS results, allowing to define chemical models explaining the data variance in the matrices and to evaluate the rate constants^28^. HS-MCR-ALS results are shown in Table 1 and Figures 3 and 4, Figure S1 (Supplementary Material).

**Table 1.** HS-MCR-ALS analysis applied to D_pHaug_ and D_oxaug_ matrices.

Data matrix	Experimental condition	k (s^-1^) x 10^-5^
**D_pHaug_**	pH 1	1.680 ± 0.025
**4 components** **LOF = 3.51%** **%R^2^ = 99.79%**	pH 3	0.023 ± 0.002
	pH 10	0.016 ± 0.002
	pH 12	k_a_ = 5.630 ± 0.032
		k_b_ = 0.740 ± 0.001
**D_oxaug_**	H_2_O_2_ and pH 1	0.004 ± 0.001
**3 components** **LOF = 3.22%** **R^2^ = 99.86%**	H_2_O_2_ and pH 7	0.106 ± 0.024
	H_2_O_2_ and pH 12	3.170 ± 0.005

**Table:** Table 2. Key protein residues of aromatase interacting with the ligands through hydrogen bonds.

Ligand	Enzyme Residues	**Distance** **Hydrogen Acceptor** **(Å)**	**Distance** **Donor-Acceptor** **(Å)**	**Angle** **Donor-Hydrogen-Acceptor (°)**
LET	Thr310	3.18	3.76	118.58
	Met374	1.92	2.87	162.87
	Ser478	1.69	2.63	162.11
DBA	Thr310	3.44	4.04	122.31
	Ser478	2.41	3.30	151.85
DBO	Thr310	3.36	3.95	121.24
	Met374	2.22	3.14	155.59
	Ser478	2.15	3.05	151.07
LNO	Arg115	2.85	3.24	104.61
	Thr310	3.10	3.94	146.23
	Met374	1.93	2.90	168.13
	Ser478	1.78	2.69	154.27

The comparison of the estimated rate constants allowed us the evaluation of the influence of pH on LTZ hydrolysis and oxidation. The drug was more affected by basic rather than acidic conditions and it was stable in an environment close to neutrality. In a basic environment, the drug degraded quickly, reaching high k values at pH 12 (k_pH12a_ = 5.63 × 1 0^−5 ^s^−1^) with a complete hydrolysis. Rapid degradation in oxidative environment was also observed, with a rate constant k_ox7_ of about 1 0^−6 ^s^−1^. Under mixed conditions, when the oxidative environment was associated with either acidic or basic pH, the stability of LTZ was reduced when the oxidative environment was coupled to a pH of 12 (k_ox12_ = 3.17 × 1 0^−5 ^s^−1^), whereas it increased at acidic pH (k_ox1_ = 4.0 × 1 0^−8 ^s^−1^).

The MCR-ALS results obtained in the analysis of the time-dependent UV data were confirmed by chromatography investigation. HPLC-DAD analysis of the samples before and after the degradation experiments confirmed the formation of the same degradation products elaborated by the MCR-ALS procedures. HPLC analysis of acid-base degradation samples provided two additional peaks of DBA (RT 6.22 min) and DBO (RT 5.81 min) in addition to the LTZ signal (RT 9.12 min), as well as a signal for the LNO product was detected during oxidative degradation (RT 7.34 min). The peaks processed by DAD had the same spectral profiles as the products resolved in the previous spectrophotometric study (see Figure S2 and S3 in Supplementary Material).

### 3.2. Docking simulations

**Figure 5.**
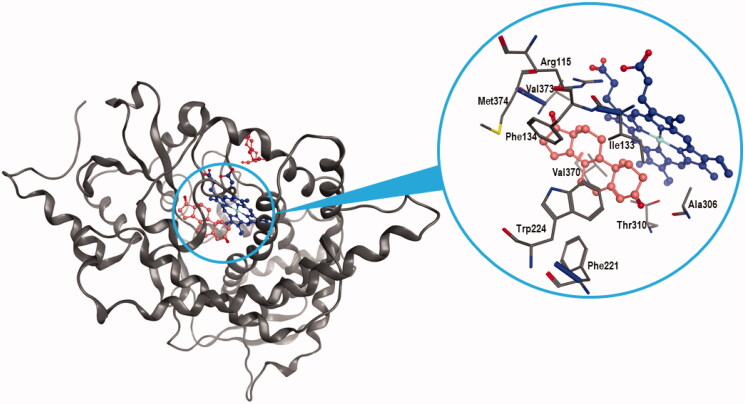
Ligand binding into the active site of aromatase. Protein backbone is represented as a ribbon. The haem group (blue), ASD (salmon), and PEG (red) are also represented. Key protein residues for the binding are labelled in the magnification.

Aromatase is a monomeric peripheral membrane protein with 503 amino acid residues, organised in 12 α-helices joined by coil regions and surrounding a haem group, as shown in Figure 5. The active site (see inset of Figure 5) lies next to a channel through which water and oxygen molecules involved in the catalysis, as well as substrates, can enter from the outer face of the membrane. At the channel entry, the couple of residues Arg192/Glu478 acts as a gate-system, whereas the inner portion of the channel ending with the binding site is characterised by the presence of both polar and ionic residues (Asp309, Thr310 and Ser478), and hydrophobic side chains (Phe221, Val313 and His480). The substrate binds at the androgen-specific haem-distal pocket, interacting with the key residues Arg115, Met374, Thr310 and Asp309. In the case of interaction with ASD, the side chains of the first two amino acids form polar contacts with the 17-carbonyl oxygen of the ligand, whereas the last two are involved in the binding with the 3-carbonyl oxygen. In particular, the protonated side chain of Asp309 seems to be critical for substrate binding orientation and catalysis^29^.

The side chain of Arg115, Ile133, Phe134, Phe221, Trp224, Ala306, Thr310, Val370, Val373, Met374 and Leu477 together with the haem porphyrin portion form a haem-distal catalytic cavity in which the ligands, including ASD, easily accommodate, interacting by means of van der Waals forces. In the aromatase structure there is also a haem-proximal cavity, located on the opposite side of the haem group with respect to the main binding site, which could host a second ligand. In the molecular complex we used as a model (PDB ID: 5JKV), this site is occupied by PEG, a pentameric fragment of polyethylenglicole, which is a weak inhibitor of aromatase able to participate in the modulation of the enzymatic activity of the protein (Figure 5). A third site acts as an access channel and connects the haem-distal binding site with the endoplasmic reticulum via the outer surface of the protein, allowing the entry and exit of steroids. Multiple additional hotspots potentially suitable to accommodate ligands have been identified within the enzyme protein, but their significance towards the protein catalytic activity remains uncertain^21^.

**Figure 6.**
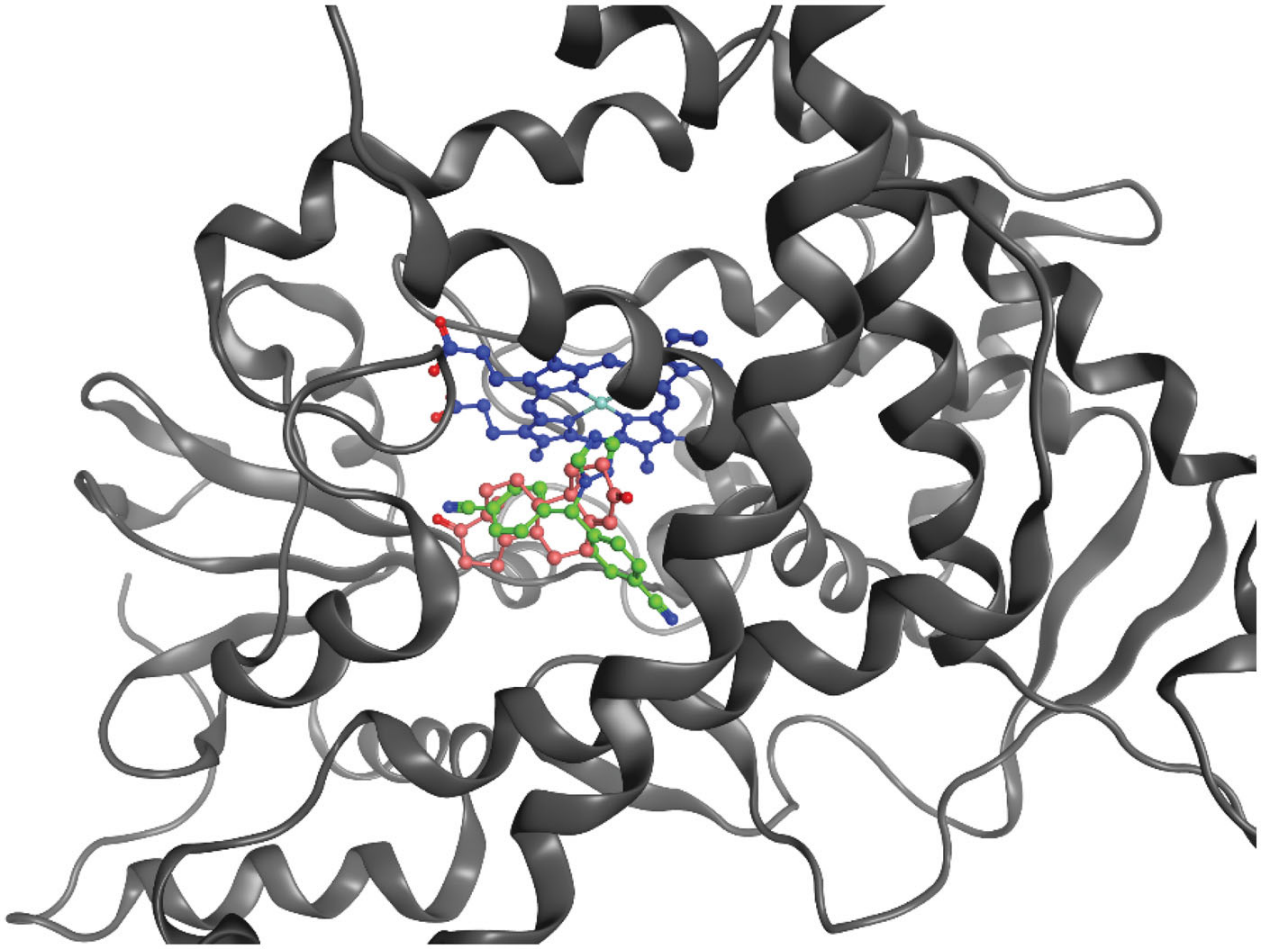
Superimposed binding mode of ASD (salmon) and LTZ (green) in the aromatase active site. The haem group is shown in blue.

A quantitative structure-activity relationship (QSAR) study^30^ predicted that the reversible aromatase inhibitors LTZ should bind the active site by forming interactions through the two cyano groups and the triazole moiety. In our docking experiments, LTZ was observed to anchor to aromatase in the main protein binding site, with a binding energy of −7.9 kcal/mol. This value corresponds to a dissociation constant in the low micromolar range, which indicates a good affinity. Furthermore, the ligand occupies the same location of ASD, as shown in Figure 6. The N_4_ atom of the 1,2,4-triazole coordinates the Fe^2+^ of the haem prosthetic group, whereas the two cyano groups of the LTZ act as a hydrogen bond acceptor by interacting with Met374 and Ser478 residues, similarly as the 3- and 17-carbonyl oxygens of the physiological ligand ASD. A further hydrogen bond forms between N_2_ atom of the triazole and Thr310. The diphenylmethane scaffold of LTZ mimics the backbone of steroids, preserving for this compound a geometry similar to that of endogenous ligands inside the binding pocket.

**Figure 7.**
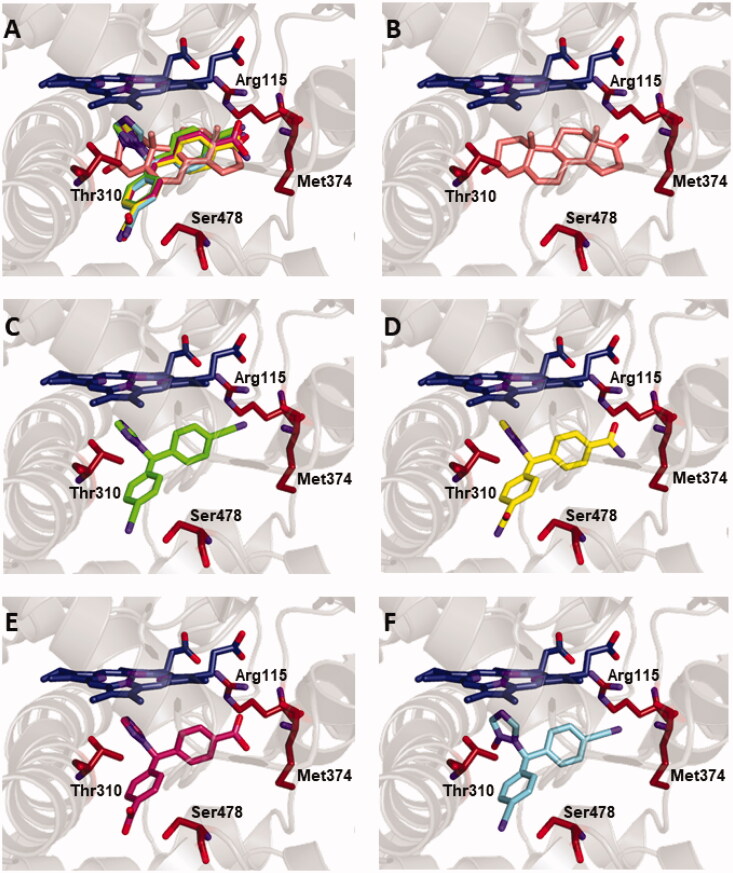
Binding of the compounds in the active site of aromatase. Protein backbone is represented as a ribbon, the haem group in dark-blue, and the key protein residues Arg115, Thr310, Met374, Ser478 in amaranth. (A) Superimposed binding modes of the physiological ligand ASD (salmon), the parent compound LTZ (green), and the degradation products DBA (yellow), DBO (pink) and LNO (cyan). The ligands are also shown separately: (B) ASD, (C) LTZ, (D) DBA, (E) DBO and (F) LNO.

Furthermore, we investigated whether the degradation compounds, deriving mainly from the transformation of the cyano groups of LTZ, could interact with aromatase in the same active site as the parent compound, and could consequently participate to the overall biological activity. Thus, LTZ degradation derivatives DBA, DBO and LNO were docked into the main catalytic site (Figure 7). In particular, as detailed in Table 2, DBO forms the same interactions as LTZ, including hydrogen bonds with the protein residues Thr310, Met374 and Ser478, whereas DBA lacks the interaction with Met374. On the other hand, a fourth hydrogen bond was observed between Arg115 and a cyano group of LNO. Hydrophobic interactions with several key residues of the site were also observed in all cases. The binding energy values observed range from −8.2 and −8.3 kcal/mol, which are slightly more favourable compared to that of the parent compound LTZ.

In summary, the simulation findings concur to predict the association of LTZ and its three main degradation products (DBA, DBO, and LNO) within the binding site of aromatase. In spite of some differences in their structural details, including charge delocalisation and ability to form hydrogen bonds, all these compounds showed clear similarities in the docking affinity and anchoring modes in the protein binding pocket. These features were evidenced in blind docking experiments, and without the need to introduce a bias to help accommodating the ligands in the protein binding site. Furthermore, the binding of these compounds is consistent with pharmacophore-based QSAR features dictated by the active site of aromatase, which may be considered an independent confirmation of our docking results. Finally, overlap in the binding modes between our compounds and the physiological ligand ASD, in spite of differences in their structure (LTZ and the degradation products assume a triangular pyramid shape, whereas ASD is a steroid with a more linear arrangement), further support the reliability of the results obtained.

## 4. Conclusions

Degradation studies indicated that LTZ, although stable to light irradiation, generates three main derivatives under stressing chemical conditions, such as changes in pH or oxidising environment. The analytical data collected by UV/Vis spectrophotometry during the kinetic experiments were processed by chemometric methodologies. The soft multivariate curve resolution of the data matrices confirmed the role of pH and oxidants in favouring the formation of the diamide (DBA), diacid (DBO) and N_2_-oxide (LNO) degradation products. The estimation of the rate constants of the degradation processes by HS-MCR showed that the parent drug is more sensitive to basic pH rather than acid or neutral pH, leading to a faster degradation and a complete transformation into the hydrolysis product DBO at pH 12 or LNO in an oxidative environment, respectively. Molecular docking experiments demonstrated that LTZ and its degradation compounds have the ability to adapt to the aromatase active site by interacting with the same key amino acid residues involved in the binding of physiological ligand ASD. The results obtained show that the metabolism of LTZ could lead to compounds still effective, suggesting that this could prolong the drug activity.

## Supplementary Material

Supplemental Material

## References

[1] Unten Y, Murai M, Koshitaka T, et al. Comprehensive understanding of multiple actions of anticancer drug tamoxifen in isolated mitochondria. Biochimica et Biophysica Acta Bioenergetics 2022;1863:1600. 10.1016/j.bbabio.2021.148520 34896079

[2] Dutta U, Pant K. Aromatase inhibitors: past, present and future in breast cancer therapy. Med Oncol 2008;25:113–24. 17973095 10.1007/s12032-007-9019-x

[3] Kharb R, Haider K, Neha K, et al. Aromatase inhibitors: role in postmenopausal breast cancer. Archiv Der Pharmazie 2020;353:2000081. 10.1002/ardp.202000081 32449548

[4] Şendur MA, Bilgin B, Hızal M, et al. Do all aromatase inhibitors have similar efficacy and safety? Future Oncol 2017;13:1673–6. 10.2217/fon-2017-0228. 28831832

[5] Annapurna MM, Mohapatro C, Narendra A. Stability-indicating liquid chromatographic method for the determination of Letrozole in pharmaceutical formulations. J Pharmac Anal 2012;2:298–305. 10.1016/j.jpha.2012.01.010 PMC5760907 29403757

[6] Elkady EF, Fouad MA. Preparation and characterization of two new forced degradation products of letrozole and development of a stability-indicating RP-LC method for its determination. Pak J Pharmac Sci 2015;28:2041–51. 26639498

[7] Ranganathan HP, Govindrajulu G, Palaniyappan V. Forced degradation study of Letrozole-A validated stability indicating HPLC assay for bulk and tablet dosage form. Inter J Pharm Pharmac Sci 2012;4:582–6.

[8] Shaban M, Ghaffary S, Hanaee J, et al. Synthesis and characterization of new surface modified magnetic nanoparticles and application for the extraction of letrozole from human plasma and analysis with HPLC-fluorescence. J Pharmac Biomed Anal 2021;193:113659. 10.1016/j.jpba.2020.113659 33176243

[9] Al-Shehri M, Hefnawy M, Abuelizz H, et al. Development and validation of an UHPLC-MS/MS method for simultaneous determination of palbociclib, letrozole and its metabolite carbinol in rat plasma and pharmacokinetic study application. Arab J Chem 2020;13:4024–34.

[10] Hegde AR, Padya BS, Soman S, et al. A simple, precise, and sensitive HPLC method for quantification of letrozole in rat plasma: development, validation, and preclinical pharmacokinetics. J Anal Sci Technol 2021;12:8.

[11] El-Kosasy AM, Rahman MHA, Abdelaal SH. Spectrofluorimetric Method for Determination of Letrozole: analytical applications to brain tissue samples and alkaline degradation kinetic study. J Appl Spectroscopy 2019;86:848–54.

[12] Acharjya SK, Mallick P, Panda P, et al. Spectrophotometric methods for the determination of letrozole in bulk and pharmaceutical dosage forms. J Adv Pharmac Technol Res 2010;1:348–53. 10.4103/0110-5558.72425 PMC3255416 22247870

[13] Ganesh M, Kamalakannan K, Patil R, et al. A validated UV spectrophotometric method for the determination of letrozole in bulk and solid dosage form. Rasayan J Chem 2008;1:55–8.

[14] Bhalla TC, Kumar V, Kumar V, et al. Nitrile Metabolizing Enzymes in Biocatalysis and Biotransformation. Appl Biochem Biotechnol 2018;185:925–46. 29380295 10.1007/s12010-018-2705-7

[15] ICH Q1A 2003. ICH Guideline Q1A(R2) stability testing of new drug substances and products. international conference on harmonization. 2003.

[16] Jaumot J, de Juan A, Tauler R. MCR-ALS GUI 2.0: New features and applications. Chem Intell Lab Syst 2015;140:1–12.

[17] De Luca M, Ioele G, Spatari C, et al. A single MCR-ALS model for drug analysis in different formulations: Application on diazepam commercial preparations. J Pharma Biomed Anal 2017;134:346–51. 10.1016/j.jpba.2016.10.022 27816253

[18] De Luca M, Ioele G, Grande F, et al. Photostability study of multicomponent drug formulations via MCR-ALS: The case of the hydrochlorothiazide-amiloride mixture. J Pharmac Biomed Anal 2020;186:113332. 10.1016/j.jpba.2020.113332 32387749

[19] Saurina J, Hernández-Cassou S, Tauler R, et al. Multivariate resolution of rank-deficient spectrophotometric data from first-order kinetic decomposition reactions. J Chemometr 1998;12:183–203.

[20] Marín-García M, De Luca M, Ragno G, et al. Coupling of spectrometric, chromatographic, and chemometric analysis in the investigation of the photodegradation of sulfamethoxazole. Talanta 2022;239:122953. 34954462 10.1016/j.talanta.2021.122953

[21] Ghosh D, Egbuta C, Lo J. Testosterone complex and non-steroidal ligands of human aromatase. J Steroid Biochem Mol Biol 2018;181:11–9. 29476820 10.1016/j.jsbmb.2018.02.009 PMC5997392

[22] Hanwell MD, Curtis DE, Lonie DC, et al. Avogadro: An advanced semantic chemical editor, visualization, and analysis platform. J Cheminform 2012;4:17. 22889332 10.1186/1758-2946-4-17 PMC3542060

[23] Trott O, Olson AJ. AutoDock Vina: Improving the speed and accuracy of docking with a new scoring function, efficient optimization, and multithreading. J Comput Chem 2010;31:455–61. 19499576 10.1002/jcc.21334 PMC3041641

[24] Morris GM, Goodsell DS, Halliday RS, et al. Automated docking using a lamarckian genetic algorithm and an empirical binding free energy function. J Comput Chem 1639;19:16391662.

[25] Grande F, Rizzuti B, Occhiuzzi MA, Statti G, et al. Identification by molecular docking of homoisoflavones from leopoldia comosa as ligands of estrogen receptors. Molecules 2018;23:894. 10.3390/molecules23040894 PMC6017050 29649162

[26] Ceramella J, Caruso A, Occhiuzzi MA, et al. Benzothienoquinazolinones as new multi-target scaffolds: dual inhibition of human Topoisomerase I and tubulin polymerization. Euro J Med Chem 2019;181:111583. 10.1016/j.ejmech.2019.111583 31400710

[27] Salentin S, Schreiber S, Haupt VJ, et al. PLIP: fully automated protein-ligand interaction profiler. Nucleic Acids Res 2015;43:W443–W447. [cited 2022 Jan 31]Available from: https://academic.oup.com/nar/article/43/W1/W443/2467865. 25873628 10.1093/nar/gkv315 PMC4489249

[28] Razuc M, Garrido M, Caro YS, et al. Hybrid hard- and soft-modeling of spectrophotometric data for monitoring of ciprofloxacin and its main photodegradation products at different pH values. Spectrochim Acta A Mol Biomol Spectrosc 2013;106:146–54. 23376269 10.1016/j.saa.2012.12.085

[29] Nardo GD, Breitner M, Bandino A, et al. Evidence for an Elevated Aspartate pK(a) in the active site of human aromatase. J Biol Chem 2015;290:1186–96. 25425647 10.1074/jbc.M114.595108 PMC4294484

[30] Nantasenamat C, Worachartcheewan A, Prachayasittikul S, et al. QSAR modeling of aromatase inhibitory activity of 1-substituted 1,2,3-triazole analogs of letrozole. Euro J Med Chem 2013;69:99–114. 10.1016/j.ejmech.2013.08.015 24012714

