# Supplementary material for: Interaction of letrozole and its degradation products with aromatase: chemometric assessment of kinetics and structure-based binding validation
Source: J Enzyme Inhib Med Chem. 2022 May 29;37(1):1600–9. doi: 10.1080/14756366.2022.2081845 (PMC9176668; doi:10.1080/14756366.2022.2081845)
Supplement: Supplemental Material [file IENZ_A_2081845_SM9685.pdf]

Supplementary

Interaction of letrozole and its degradation products with aromatase: chemometric assessment of kinetics and structure-based binding validation

Michele De Luca*, Maria Antonietta Occhiuzzi, Bruno Rizzuti, Giuseppina Ioele, Gaetano Ragno, Antonio Garofalo, Fedora Grande*

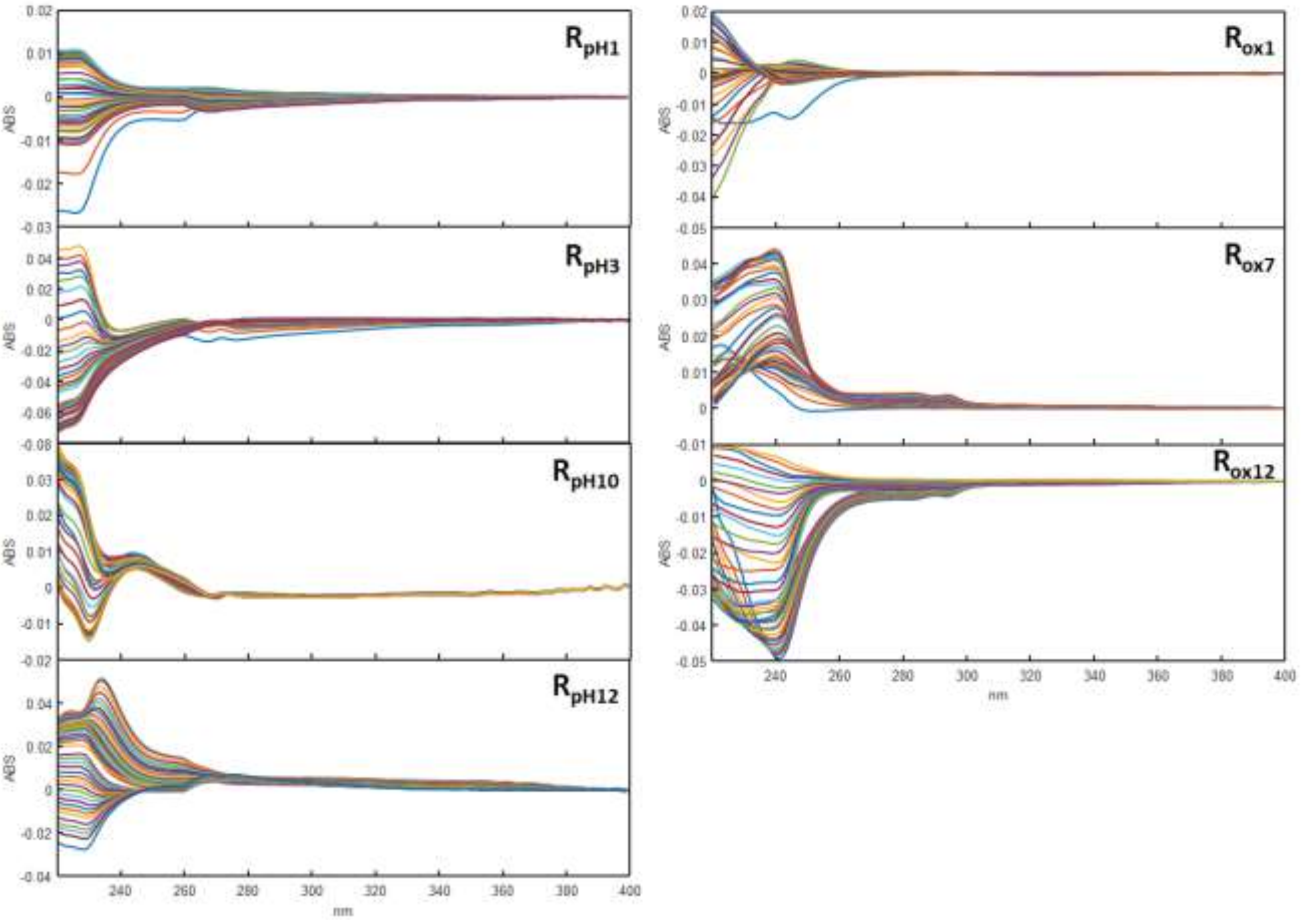

Figure S1. Residual profiles calculated in HS-MCR-ALS resolution for acid-base (**$R_{pH\ 1,\ 3,\ 10,\ 12}$**) and oxidative (**$R_{ox\ 1,\ 7,\ 12}$**) conditions.

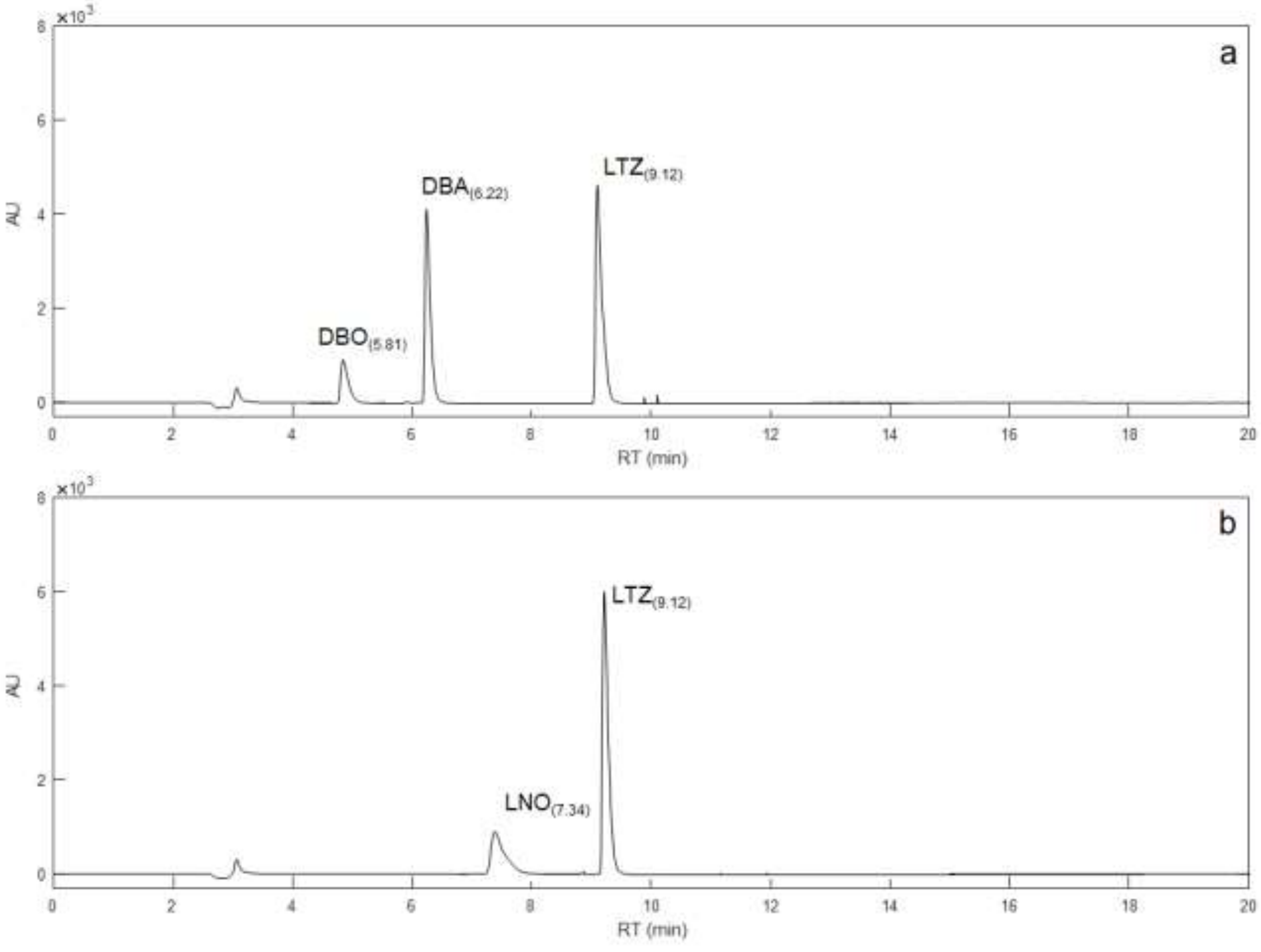

Figure S2. Letrozole and degradation products analysed by HPLC runs after (**a**) 6 h degradation at pH 12 and (**b**) 8 h degradation in oxidative conditions.

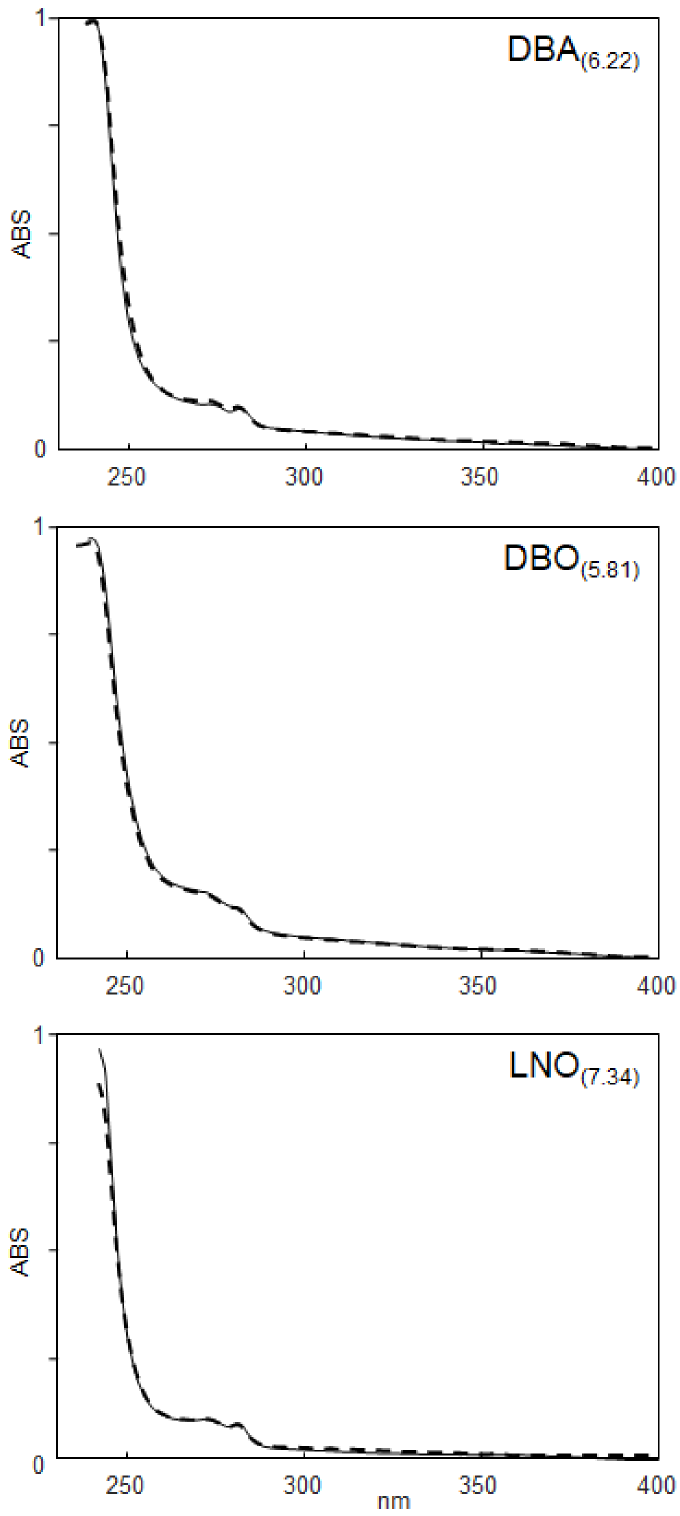

Figure S3. Comparison of the spectra obtained by MCR resolution of spectrophotometric data (dotted line) and DAD-HLPC signals (solid line).
